# Development and application of an urban solar photovoltaic opportunity mapping tool

**DOI:** 10.1016/j.heliyon.2024.e32123

**Published:** 2024-05-29

**Authors:** R. McGhee, J.A. Clarke, K. Svehla

**Affiliations:** Energy Systems Research Unit, Mechanical and Aerospace Engineering, University of Strathclyde, James Weir Building, 75 Montrose Street, Glasgow, G4 0NG, UK

**Keywords:** Solar PV, Urban opportunity mapping, Policy/technical rating, Site suitability, QGIS

## Abstract

A Geospatial Opportunity Mapping (GOMap) tool was created to identify policy unconstrained land in urban cities that is technically feasible for the deployment of solar photovoltaic power stations; and identify buildings with north- or south-facing orientation for the installation of building integrated PV (BIPV). Collaboration with a local Governing authority and a local electricity provider enabled the process to elicit comprehensive policy and technical aspect information respectively that would impact the site selection process. Five policy and four technical aspects are comprised of a total of 36 individual factors displayable by GOMap on a high-resolution city grid with a scoring system implemented to distinguish between factors that encourage or inhibits solar PV deployment. Weightings can be applied, and different scenarios explored including alternative policy changes and infrastructure upgrades. GOMap generates opportunity maps in the form of available land estimates which can be extrapolated by an in-built solar PV model to quantify annual energy generation based on local weather data, array spacing, panel type and array tilt angle. Three scenarios were devised to identify unconstrained land for solar PV deployment with varying levels of policy and technical factor relaxation, and a fourth scenario to identify dwellings for potential BIPV. These scenarios aim to tackle Glasgow City's growing energy demand and fuel poverty issue, the latter of which can supply energy to dwellings categorised as ‘hard-to-heat’ once heating is electrified due to the Scottish Government's Energy Strategy commitment.

## 1 Introduction

Climate change has become a scientific and political hot topic where the mitigation of carbon emissions is considered a top priority with investments poured into renewable energy projects around the World [[1], [2], [3]]. As society has become technology-dependent with consumers regularly purchasing new electrical devices and manufacturers frequently producing more energy efficient electrical goods [4,5], energy consumption was seen to decrease during certain years and rebound in other years particularly during the COVID-19 pandemic where much of the population around the world worked and studied from home [6]. It is necessary to ensure there is energy security, whilst at the same time, ensuring targets to reduce energy consumption are being met. The European Union has implemented Directive 2018/2001, aiming to promote renewable energy use, requiring 32 % of energy consumption to come from renewable sources by 2030 [7]. There is also the growing realisation that renewable energy technology (RET) deployment need not be restricted to strategic power production but may also be pursued at the intra-urban level to facilitate a better demand-supply match [8]. The EU Horizon 2020 programme has funded several industry/academic collaborations to explore the possibilities of urban low carbon energy solutions [9]. Sustainable cities are being driven by projects like intelligent street lighting, smart transport, and wireless networks for improved energy efficiency [[10], [11], [12], [13]].

Renewable energy is paving the way for clean energy generation as various low-carbon solutions can be installed across rural and urban landscapes and generate energy that can be used by communities or injected into a national electrical grid network [14]. However, land assessments such as on-site surveys would need to be conducted to confirm whether any RET can be deployed with relative ease at a given location as these sites may be affected by issues making it policy or technically difficult for the site to undergo any kind of development [15]. These issues can include negative impacts on the local environment and biodiversity [16], poor ground conditions and inadequate infrastructure [17], or insufficient energy generation potential [18]. Additionally, these issues are commonly recorded on a spatial format that allows Geographic Information Systems (GIS) to visualise the issues on a digital map. GIS technology is a crucial decision support tool for spatial analysis and planning, enabling pre-defined criteria and constraints to be computed and visualized before decisions are made by identifying policy and technical issues that are required to be managed or mitigated [19,20]. GIS technology could be used to develop mapping tools for identifying site suitability for RET deployment, but many focus on large regions and single issues, lacking information on other significant aspects. For example, a project in Spain investigated the deployment of solar PV on land where proximity to the nearest connection point to the grid network was weighted heavily [21]. Other aspects taken into consideration were weather for solar irradiance, terrain for the sloping angle of the land, and the environment. However, it was noted by the authors that only a single environmental factor was considered which related to the agrological soil capacity as this project was in its initial phase. Another solar PV deployment project in Iran weighted weather heavily with few other aspects considered including proximity to cities and roads, terrain, and the environment, the latter of which was limited and focused on the Normalized Difference Vegetation Index (NDVI) to protect agricultural land [22]. From these and various other projects [[23], [24], [25], [26], [27]], there is limited information used for aspects which should be comprehensive during the analysis. The environmental aspect alone encompasses a wide range of factors: from conservation areas to protected areas of scientific interest; from tree preservation to historic monuments. This level of detail may not exist in some project areas, or it may not be readily available, but this leads to the need that when evaluating potential sites for RET deployment, it is vital to include the local authorities and local utility providers as these bodies would likely possess the relevant information necessary and decide if such a deployment can proceed based on policy regulations and technical feasibility. In addition, policy and technical aspects described in these projects were weighted according to literature reviews conducted by the authors. However, there was no standard for prioritizing similar aspects as the importance of these differ from country to country and city to city. As a result, some projects give higher significance to certain aspects over others. That said, there have been some projects where the weightings have been derived by researchers following discussion with stakeholders and policymakers [[28], [29], [30]]. Ideally, this collaboration should be a common method when assigning weightings. The GOMap tool was designed in collaboration with such bodies and its development will now be discussed in the next section. The Future City Demonstrator Project [31], funded by Innovate UK, was a significant initiative in the development of future cities and commissioned the Energy Systems Research Unit (ESRU) to develop a new evaluation method encapsulated in a GIS tool that captured and analysed policy aspects from a local planning authority, and technical aspects from a local utility provider in spatial format. The research project resulted in the creation of GOMap, a Geospatial Opportunity Mapping tool [32], built on top of the open-source QGIS framework [33]. The tool provides feedback on solar photovoltaic (PV) panel performance and local policy regulations, and is made publicly available under an open-source license. It identifies city areas where community-scale renewable energy schemes can be most easily deployed. A significant innovation of the tool allows users to modify input data such as switching on/off any spatial information or shifting the weightings where such modifications would cause the final opportunity map to be recalculated in real-time. Compared to other linear models used by some of the aforementioned projects, the GOMap tool performs all geoprocessing operations during the importation of spatial data. Once all data has been imported and the opportunity map generated, any changes made in the interface causes the opportunity map to recalculate and refresh. The tool can also be reconfigured to identify buildings with north- or south-facing rooftops and investigate the benefit of installing solar PV panel arrays for maximum solar energy gain.

This paper is based on the author's doctoral thesis [34] and focuses on the core development of the GOMap tool itself, its application on identifying prospective land sites within a city, its application on identifying buildings with south-facing rooftops, and the numerical results obtained from this analysis. Two sister journal papers were published: the first paper introduced the GOMap tool, the types of information required, and its application to identify vacant and derelict land (VDL) sites for the deployment of solar PV panels [35]; the second paper described how the GOMap tool can be expanded for other RETs such as wind turbine siting and identifying suppliable dwellings within a local district heating network [36].

This paper is divided into five sections. Section 2 describes the required framework of GOMap and its development to facilitate its ability to identify new energy site generation. Section 3 describes the solar PV modelling and the tool's ability in identifying suitable dwelling rooftops for BIPV. Section 4 describes the application and results of the tool's search functionality for new energy site generation. And Section 5 summarises the findings and provides concluding remarks.

## 2 GOMap framework

GOMap is built upon the open-source QGIS software package supporting a wide range of extensions by allowing direct source-code modification. GOMap is a Python-based source-code (version 3.7), developed using Qt designer (version 5.15), and compatible with QGIS (version 3.28 or later).

There are three main constituents of GOMap: the grid system to capture and store all spatial information on a variable resolution; the scoring system to differentiate policy and technical information that support or curtail renewable energy deployment; and the weighting system to prioritise policy and technical information based on city planning procedures and expert knowledge. Automated scripts are utilized to import and adjust spatial information to a local coordinate reference system [37].

### 2.1 Spatial information

The digital storage of spatial information has led to the creation of Shapefile, a geospatial vector data format specifically designed for GIS software [38]. Vector features such as points, lines, and polygons, can be produced and contained within a shapefile where each feature can store attribute data in the form of a database. Local authorities maintain their catalogue of shapefiles to represent information including those related to biodiversity and city development; similarly, utility providers hold information pertaining to electrical substations and power cable networks [35]. External information can be collated to develop a wealth of information stored in spatial format. Information held in other formats such as digital raster models are vectorised to ensure compatibility with all other information.

### 2.2 Grid system

**Fig. 1:**
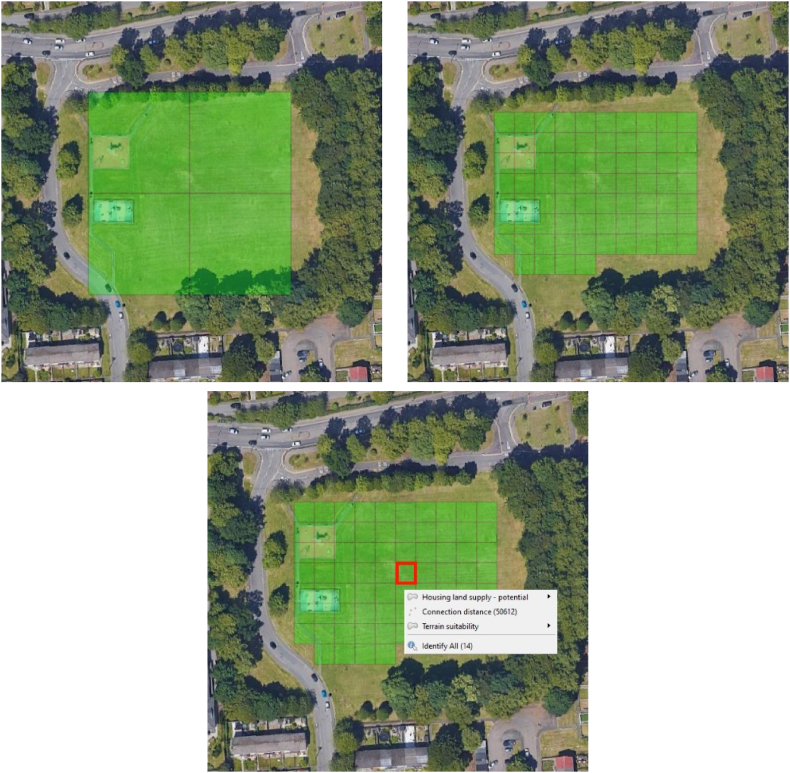
Varying grid resolutions: low (top-left) and high (top-right). The images show grid cells which cover the site at different resolutions. Clicking on a cell shows which policy/technical information are present (bottom-centre).

The grid system is a vital foundation of GOMap's opportunity mapping analysis and has two significant roles. The first role allows the grid resolution to be defined at any scale and cover the full extent of the city being investigated. If the grid resolution is too low, it can cause the grid cells to overlap unnecessary areas of the perimeter of a site which may contain access roads. Therefore, the grid system is equipped with a variable overlap rule that is set at a default value of 50 %. Now, if a grid cell occupies less than the defined overlap percentage, it is removed from the analysis and the opportunity map. Therefore, cells should ideally be within the site to allow for a more accurate analysis. The top two images in Fig. 1 show an example of two grid resolutions applied to a site with the top-left image consisting of a low-resolution which overlaps surrounding trees and a small section of a road. As most of the cell falls within the site, it is displayed in the opportunity map even though parts of the cells are constrained by trees and a road; the top-right image depicts a higher resolution grid where most of the cells is contained within the site and as a result, GOMap can remove those cells which are constrained by trees and a road.

The second role of the grid system facilitates the import of policy and technical spatial information to be contained within the grid cells. Each cell is assigned a unique ID allowing the user to focus on individual policy and technical information affecting the cell as shown in the bottom-centre image in Fig. 1.

The generation of the grid system is achieved by running a Shapefile Conversion Script which reads input spatial information, fragments this input into grid cells, and ensures the grid cells from different policy and technical information possess the corresponding ID based on the local coordinate system to confirm consistency. In addition, GOMap enables the creation of projects with the same spatial information at different resolutions where a low-resolution opportunity map can be used for testing or demonstration purposes; whilst a high-resolution opportunity map can be used for in-depth analysis.

### 2.3 Factor scoring

Policy and technical spatial information are categorised into Aspects. Policy aspects can include but is not limited to biodiversity, city development, environmental, and social. Similarly, technical aspects can include but is not limited to: terrain, proximity to transport links, proximity to the electrical grid network, and weather. Each of these aspects can be further decomposed into Factors. An example is the environmental aspect which can consist of several factors such as Local nature reserves, World Heritage Sites, etc.

To complement the grid system, each factor is assigned a score which emerged from workshops with local authority planners and utility specialists and from the study of planning documentation. A 3-point scoring was established for simplicity and to avoid subjectivity as shown in Table 1 where lower scores encourage project development and higher scores curtail it. For extreme cases, a showstopper score is included where a factor underpinned by a policy aspect cannot be mitigated. Such factors can occur in rare cases such as those related to the environment or World Heritage Sites.

**Table 1:** Policy and technical factor scoring [34].

Score	Policy	Technical
1	Possible	Favorable
2	Intermediate	Likely
3	Sensitive	Unlikely
4	Showstopper	–

With the implementation of the grid system, a scoring mechanism is designed to take advantage of the unique IDs for each grid cell. This aids in the evaluation of each factor and enables the calculation of the overall score for each cell. Two methods of scoring are incorporated into the tool: the lenient method which calculates either the median or median of all individual factor scores for each aspect; and the stringent method which assigns the highest overlapping factor score. The former method became the default setting of the tool to support planners and developers to explore sites for renewable energy deployment.

### 2.4 Aspect weighting

Due to the numerous aspects related to policy and technical considerations, it was vital to establish a procedure to prioritise the aspects considered more significant compared to others. This weighting system allows for available resources to be screened based on all policy and technical information provided and encourages investigating alternative policy changes and infrastructure upgrades. This feature turns the GOMap tool into a GIS MCDM support tool to tackle complex problems and provide solutions based on multiple criteria [23].

This support is extended further by the Analytical Hierarchy Process (AHP) method developed by Saaty [39] as a process to resolve *n* criteria using pairwise comparison matrix *A* (*n x n*) for aspects, *a*, as expressed in equation (1):

(1) $$A=\left[a_{ij}\right]=\left[\begin{array}{c} \begin{array}{cc} a_{11} & a_{12}\\ a_{21} & a_{22}\\ \cdots & \cdots \end{array}\;\begin{array}{cc} \cdots & a_{1n}\\ \cdots & a_{2n}\\ \cdots & \cdots \end{array}\\ \begin{array}{cc} a_{n1} & a_{n2} \end{array}\;\begin{array}{cc} \ldots & a_{nn} \end{array} \end{array}\right]$$

where *a*_*ii*_ presents the importance of *i*_*th*_ criterion to the *j*_*th*_ criterion.

Within the GOMap tool, AHP is applied to calculate the most suitable weightings for all policy and technical aspects for a given scenario. This method also safeguards the consistency of the final weightings produced by removing subjective bias during the decision-making phase [40]. A scale from 1 to 9 is used to define the relative importance between aspects where larger numbers imply greater importance and the value of 1 implying equal importance. A reciprocal scale from −1 to −9 is also used to define the relative importance between aspects where smaller numbers imply lesser importance. Two weighted matrices are generated – one for all policy aspects, and another for all technical aspects.

To determine the most suitable weightings, the first step is to normalise the pairwise comparison matrix by summing the values in each column of the ratio matrix as expressed in equation (2) [41]:

(2) $$a_{ij}=\sum_{i=1}^{n}a_{ij}$$

where *n* denotes the number of aspects.

The matrix is normalized pairwise by dividing each element by its column total within it, *X*_*ij*_ using equation (3) [41]:

(3) $$X_{ij}=\frac{a_{ij}}{\sum_{i=1}^{n}a_{ij}}\left[\begin{array}{c} \begin{array}{cc} X_{11} & X_{12}\\ X_{21} & X_{22}\\ \cdots & \cdots \end{array}\;\begin{array}{cc} \cdots & X_{1n}\\ \cdots & X_{2n}\\ \cdots & \cdots \end{array}\\ \begin{array}{cc} X_{n1} & X_{n2} \end{array}\;\begin{array}{cc} \ldots & X_{nn} \end{array} \end{array}\right]$$

The sum of each normalized column is divided by the number of aspects used to create the final weighted matrix, *W*_*ij*_ using equation (4) [41]:

(4) $$W_{ij}=\frac{\sum_{i=1}^{n}X_{ij}}{n}\left[\begin{array}{c} W_{11}\\ W_{12}\\ \cdots \\ W_{1n} \end{array}\right]$$

To ensure reliability and the removal of any subjective bias, a Consistency Ratio (CR) is determined in equation (5) [42]:

(5) $$CR=\frac{CI}{RI}$$

where CI is the Consistency Index and RI is the Random Index.

CI is a measure of inconsistency to evaluate the consistency of decisions made when scaling aspects. CI is 0 when either all judgments are perfectly consistent, or n < 3, and expressed in equation (6) [40,41]:

(6) $$CI=\frac{\lambda -n}{n-1}$$

where λ denotes the average of the consistency vector, C_v_, as seen in equation (7) [43]:

(7) $$\lambda =\sum_{i=1}^{n}C_{v}$$

C_v_ is determined by dividing the sum of the weightings within each column of the ratio matrix by the criterion weights.

RI is the consistency index of a randomly generated pairwise comparison matrix of order 1–10. This index was generated by approximating random indices using a sample size of 500 [44].

If CR < 0.1, the result shows acceptable consistency in the pairwise comparisons and the aspect weightings can be used for generating opportunity maps. However, if CR > 0.1, then the results are inconsistent, and the importance scales assigned to each aspect would need to be revisited [44].

Once the weightings have been determined, the final score of a grid cell, *S*, is calculated by multiplying each aspect score, *A*, by the aspect's weight, *W,* and taking the final sum as expressed in equation (8) [45]:

(8) $$S=\sum_{i=1}^{n}W_{i}A_{i}\;\text{.}$$

Alternatively, equal weightings can be set where all policy aspects are treated with equal importance, similarly with all technical aspects. This allows planners to devise simple or base-case scenarios which can be useful when comparing to scenarios with defined weightings normally derived from consultations with policymakers, utility experts and stakeholders [46,47].

## 3 Renewable energy modelling

### 3.1 Solar photovoltaic

A solar PV model has been built into GOMap based on equations related to solar geometry and solar irradiance which allows for site coordinates to be passed into the PV model to calculate the hourly power output of an array of PV panels.

The solar geometries are determined by calculating the declination angle of the Sun, *d*, which is the angle between the equator and a line drawn from Earth to the sun, and *y* is the year day number as expressed in equation (9) [48]:

(9) d=23.45sin(280.1+0.9862y)

The Earth's orbit's eccentricity and axial tilt are corrected using the equation of time, *EoT*, as shown in equation (10) [49]:

(10) EoT=9.87sin(1.978y−160.22)−7.53cos(0.989y−80.11)−1.5sin(0.989y−80.11)

The local solar time, *t*_*s*_, adjusts for the longitude where *GMT* is the Greenwich Mean Time and L is the longitude difference as seen in equation (11) [50]:

(11) $$t_{s}=\text{GMT}+L/15+\text{EoT}/60$$

The hour angle, *θ*_*h*_, converts *t*_*s*_ to an angle relative to the local reference taking into consideration that each hour from solar noon, the Sun travels approximately 15^o^, as expressed in equation (12) [50]:

(12) $$\theta_{h}=15\;*\;\left(12\;-\;t_{s}\right)$$

The solar elevation denoted by *β*_*s*_ is the angular height of the Sun as can be seen in equation (13) [50]:

(13) $$\beta_{s}=\sin^{-1}\left(cosL\;*\;cosd\;*\;\cos\;\theta h+sinL\;*\;sind\right)$$

The solar azimuth, *α*_*s*_, is the horizontal angle of the Sun measured from the North in the northern hemisphere shown in equation (14) [50]:

(14) $$\alpha_{s}=\sin^{-1}\left(cosd\;*\;\sin\;\theta_{h}\;/\;\cos\;\beta_{s}\right)$$

The wall-solar azimuth, denoted by *ω*, is the difference between the solar and surface azimuths, with the latter being denoted by *α*_*f*_, as expressed in equation (15) [50]:

(15) $$\omega =\alpha_{s}\;-\;\alpha_{f}$$

The final component of the solar geometry to calculate is the solar incidence angle, *i*_*β*_*,* between the normal of the solar PV panel surface and the direct vector of the Sun where *β*_*f*_ is panel tilt angle, expressed in equation (16) [51]:

(16) $$i_{\beta }=\cos^{-1}\left(\sin\;\beta_{s}\;*\;\cos\left(90\;-\;\beta_{f}\right)+\cos\;\beta_{s}\;*\;\cos\;\omega \;*\;\sin\left(90\;-\;\beta_{f}\right)\right)$$

With the solar geometries defined, the solar irradiance could then be determined which constitutes three individual parts – the first of which is the direct solar irradiance, *I*_*dh*,_ where *I*_*dβ*_ is the horizontal component of the irradiance as shown in equation (17) [52]:

(17) $$I_{d\beta }=I_{dh}\;\cos\;i_{\beta }\;/\;\sin\;\beta_{s}$$

The second part of the solar irradiance is the sky diffusion, *I*_*sβ*_, based on anisotropic brightness distribution where *I*_*fh*_ represents diffuse horizontal irradiance, while *I*_*Th*_ represents the sum of direct and diffuse horizontal irradiance as shown in equation (18) [53]:

(18) $$I_{s\beta }=I_{\text{fh}}\;*\;\{0.5[1+\cos\left(90\;-\;\beta_{f}\right)]\}\;*\;\{1+[1\;-\;\left(I_{fh}^{2}\;/\;I_{Th}^{2}\;\right)]\sin^{3}\;\left(0.5\;\beta_{f}\right)\}\;*\;\{1+[1\;-\;\left(I_{fh}^{2}\;/I_{Th}^{2}\;\right)]\cos\;2i_{\beta }\;\sin^{3}\;\left(90\;-\;\beta_{s}\right)\}$$

The third part is the irradiance due to ground reflection, *I*_*rβ*_, where the ground reflectance is denoted by *r*_*g*_ as shown in equation (19) [50]:

(19) $$I_{r\beta }=0.5\left[1\;-\;\cos\left(90\;-\;\beta_{f}\right)\right]\left(I_{Th}\right)r_{g}$$

The summation of all three irradiance parts is given by *I*_*total*_ and expressed in equation (20):

(20) $$I_{total}=I_{d\beta }+{I_{s\beta }+I}_{r\beta }$$

The power output of a solar PV array is estimated from the following equation which includes the power output under Standard Test Conditions, *P*_*STC*_; an empirical coefficient, *β*; the operating temperature in degrees Celsius, *T*; and the number of panels, *p*, as shown in equation (21) [54]:

(21) $$Power=P_{STC}\;\left(\frac{I_{total}}{1000}\right)\left(1-\beta \left\{T-25\right\}\right)*p$$

The deployment of solar PV arrays necessitates a minimum distance between the rows of panels to prevent any shade from nearby rows. The spacing of panels is determined as a function of panel length, *L*, tilt angle, *θ*_*t*_, and the Sun elevation angle, *θ*_*e*_, the latter of which is averaged across the seasonal equinoxes and solstices. The array spacing, *D*, consists of the horizontal component of the tilted PV panel, *D*_*1*_, and the horizontal component based on the tilt angle and the solar altitude, *D*_*2*_, expressed in equation (22) [55,56]:

(22) $$D=Lcos\theta_{t}+L\frac{\sin\;\theta_{t}}{\tan\;\theta_{e}}\;\text{.}$$

As the Sun's elevation angle is averaged throughout the year, some overshading will occur during the Winter months. To avoid overshading entirely would entail fewer rows of solar PV panels which, although this would be beneficial in Winter, would be a wasted opportunity during Summer. Therefore, a reasonable balance was necessary. Additionally, overshading can occur due to tall structures such as buildings and trees. In this instance, the horizontal distance of a structure's shadow, *D*_*shadow*_, can be calculated using the height of the structure, *h*_*structure*_, and the Sun elevation angle, *θ*_*e*_, expressed in equation (23) [57]:

(23) $$D_{shadow}=\frac{h_{structure}}{{\tan(\theta }_{e})}$$

Here, the structure's shadow was calculated for each solstice and equinox, which were superimposed to provide a composite annual overshading footprint. Although calculating the overshading of buildings is not part of the solar PV model, it is used to generate the overshading spatial dataset which would discount any areas of land which fall within this dataset.

The amount of unconstrained land utilized for solar PV array deployment can be controlled via the GOMap's Land Utilisation option which allows for a proportion of the land to be available for deployment whilst ensuring the remaining proportion is available for utilisation for other means. The default value for land utilisation is 50 % as studies have shown a fixed-tilt PV farm utilise between 47 % and 51 % of available land [[58], [59], [60]].

The solar PV model uses GOMap-identified unconstrained site areas to generate polygon features for PV panels on the opportunity map. It determines the number of panels for each site based on geometry coordinates and converts this into annual energy yield predictions based on annual power output. The model allows for input of known solar PV installation parameters, otherwise optimal settings are automatically calculated based on local weather information and site coordinates.

### 3.2 Building integrated photovoltaics

The initial configuration of GOMap focused on available land, or more specifically, land designated as vacant and/or derelict. This section describes how the tool can be reconfigured to focus on BIPV, i.e. the effects of installing solar PV arrays on the rooftops of occupied dwellings.

Identifying whether a rooftop faces the northerly or southerly direction is significant regarding solar PV installation as locations based in the northern hemisphere would benefit immensely from PV arrays facing the southerly direction. There are various processes which can be used to extract rooftop footprints including: height and colour-infrared information from a Digital Surface Model (DSM) in conjunction with NDVI to recognise surfaces of roofs [61]; exploiting a Digital Elevation Model to exclude areas below a specified height [62]; and utilising a DSM filtered over a building polygon layer to obtain the rooftop geometry [63].

**Fig. 2:**
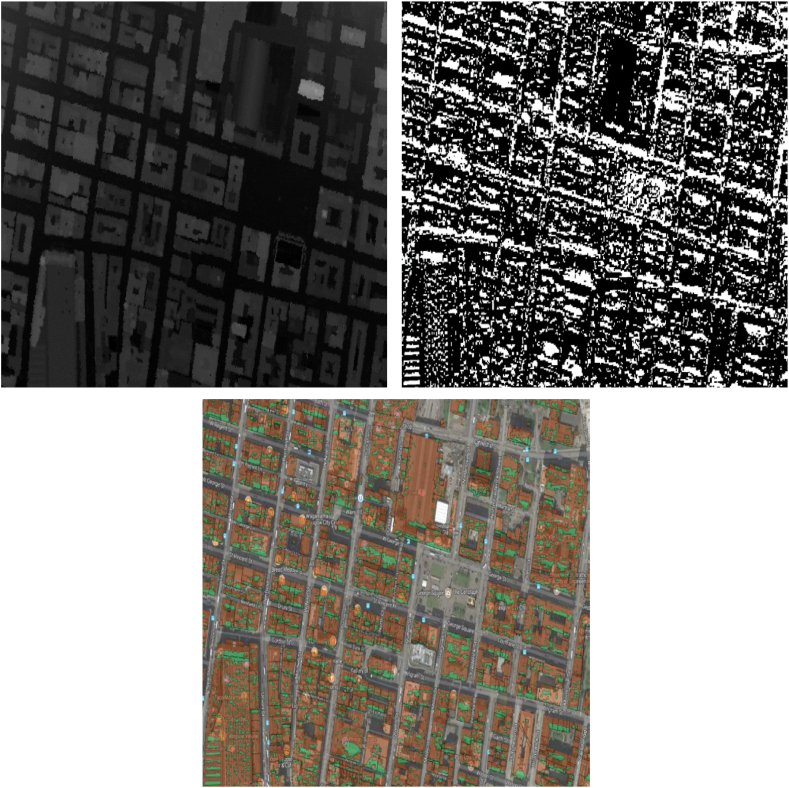
Procedure to identify southerly-facing rooftops: Original DSM (top-left); Model converted to a raster file (top-right); Raster reclassified to Boolean values depicted as south-facing (green) and superimposed with building polygons (orange) (bottom-centre).

For the GOMap tool, the latter process of utilising a DSM with a building polygon layer is implemented to identify and capture building rooftops facing either the northerly or southerly direction. This method was chosen as it works efficiently with polygon datasets which allows for effective interaction with GOMap. The DSM (top-left image of Fig. 2) contains height information for the entire coverage of a city including tall structures such as trees and buildings; and the second which is a building polygon shapefile contains the geometry of the roofs for each building and whether the building itself is for commercial or living purposes such as offices, flats, or dwellings. A high-resolution DSM would ensure the analysis is conducted accurately and discount areas of rooftops which are not fully exposed to solar irradiance and thus, would not be beneficial for solar PV deployment [64].

The DSM is converted into an image raster file with each pixel storing a value representing its directional angle with respect to its neighbouring pixels (top-right image of Fig. 2). A pixel with value 0^o^ faces due north; value 90^o^ faces east; and so on. All pixels are reclassified to Boolean values where pixels facing the southerly direction (ranged between 135^o^ and 225^o^ from due north) are given the value 1; all other pixels are given the value 0 and consequently removed from the raster. The remaining pixels are spatially intersected with the building polygon shapefile, resulting in a new output shapefile with south-facing rooftop polygons (bottom-centre image of Fig. 2).

Dwellings can be selected, and its southerly-facing roof area transferred to the solar PV model to calculate the number of arrays that could be physically installed on the roofs considering the sloping angle for slanted roofs and the energy potential of these arrays.

## 4 GOMap application

### 4.1 Consultation

Glasgow City Council won funding from Innovate UK as part of the Future City Demonstrator Project to convert its VDL for deployment. Land designated as vacant and/or derelict is a widespread issue as the city has the third highest amount of such land in all Scottish cities, comprising a total of 880 ha throughout 644 sites. Glasgow alone accounts for 9 % of all VDL in Scotland and as a result, the City Council are wanting to redevelop VDL for economic utilisation by attracting new businesses and providing job opportunities [65]; build new housing to tackle Glasgow's homeless issue [66]; and deploying RET to supply the city's energy demand [67], the latter of which was the focus of this project.

Comprehensive spatial information was obtained from relevant bodies: the local authority (Glasgow City Council) for policy information and the local utility provider (Scottish Power Energy Network) for technical information. Based on a parametric study [34], a 10 m × 10 m grid resolution was set for Glasgow City. This resolution consists of 1.76 million cells and was found to be more than sufficient for a thorough analysis.

During consultations held with both bodies, five policy and four technical aspects including their significance were identified.

The five policy aspects are designated as follows:

• Biodiversity – The Nature Conservation (Scotland) Act 2004 significantly protects biodiversity, highlighting its high significance in preserving it [68].
• Developmental – its significance was low, as it aimed to strategically use land for economic and social growth through industrial infrastructure expansion and housing attraction, compared to addressing biodiversity and environmental protections set in legislation.
• Environmental – its significance was equivalent to biodiversity and crucial for protecting natural green areas and preserving landscapes, as outlined in legislations such as the Planning (Listed Buildings and Conservation Areas) (Scotland) Act 1997 [69], The Sites of Special Scientific Interest Regulations 2008 [70], and UNESCO World Heritage Sites.
• Visual impact – its significance was considered moderate as close proximity of built-up areas to a solar PV farm can cause friction in communities due to an impact on local aesthetics which may increase the risk of vandalism.
• Visual intrusion – its significance was deemed minimal due to the low risk of glare endangerment within aerodrome traffic zones from solar PV panels as no major incidents has been reported from airports with local PV systems.

The four technical aspects are designated as follows:

• Overshading – its significance was considered very high as it can significantly reduce power output from tall structures like trees and buildings, as the amount of solar irradiance received is crucial.
• Substation congestion – its significance was considered low as it could improve over time with infrastructure upgrades.
• Substation connection distance – its significance was considered moderate to prevent unnecessary excavation of ground or fields, which could negatively impact traffic, local communities, and the environment.
• Terrain – its significance was deemed minimal due to solar PV panels' lack of deep foundations, environmental impact, and the potential for flood mitigation to be implemented.

Accommodating the MCDM/AHP method in conjunction with city planning policies [71,72] from the Land and Environmental Services department in Glasgow City Council and energy experts from Scottish Power Energy Network, produced the final weightings of all policy and technical aspects as shown in Table 2, Table 3 respectively. These tables also show factor information associated with each aspect such as the score and a brief description. In total, 36 factors were composed and contained within the aforementioned aspects with extensive details reported elsewhere [34,35]. No weather aspect was included as such information would be embedded in GOMap's renewable energy modelling capabilities.

**Table 2:** Policy aspect and factor information [34].

Aspect	Weighting	Rating	Score	Factor
*Biodiversity*	0.326	Possible	1	The protected list does not contain any species that are believed to be present.
		Intermediate	2	A UK protected species may be resident, necessitating an environmental survey and mitigation measures.
		Sensitive	3	A European protected species may be residing in the area, necessitating an environmental survey and significant mitigation measures.
*Developmental*	0.114	Possible	1	Masterplan; Strategic economic investment locations; Transformational regeneration.
		Intermediate	2	Community growth masterplan; Economic policy; Green belt; Green network opportunity; Housing land supply; Industrial-business marketable land supply; Local development framework; Network of Centers; Strategic development framework; Strategic development framework - river.
		Sensitive	3	Housing land supply with consented developments.
*Environmental*	0.326	Possible	1	Green corridors; Local nature reserves.
		Intermediate	2	Ancient woodlands; Conservation areas; Listed buildings; Tree preservation orders; World Heritage Site buffer zone.
		Sensitive	3	Gardens and designed landscapes; Scheduled ancient monuments; Sites of importance for nature conservation; Sites of special landscape importance.
		Showstopper	4	Sites of Special Scientific Interest; World Heritage Site (Antonine Wall).
*Visual impact*	0.148	Possible	1	Site not viewed from any residential areas ( ≥ 500 m)
		Intermediate	2	Site viewed from a residential area (<500 m).
*Visual intrusion*	0.086	Possible	1	All other areas.
		Intermediate	2	Between 1 km and 5 km radius of an airport/heliport or within 100 m of a motorway.
		Sensitive	3	Within 1 km radius of an airport/heliport or within 100 m of a motorway.
*Σ*	1.000	–	–	–

**Table 3:** Technical aspect and factor information [34].

Aspect	Weighting	Rating	Score	Factor
*Overshading*	0.484	Favorable	1	Outside the estimated annual shaded footprint.
		Unlikely	3	Within the estimated annual shaded footprint.
*Substation congestion*	0.168	Favorable	1	Heat map congestion score under 10.
		Likely	2	Heat map congestion score equal to 10.
		Unlikely	3	Heat map congestion score greater than 10.
*Substation connection distance*	0.231	Favorable	1	Within 100 m of a substation connection line.
		Likely	2	Between 100 m and 200 m of a substation connection line.
		Unlikely	3	Further than 200 m from a substation connection line.
*Terrain*	0.117	Favorable	1	No risk of flooding, no access issues, or flat ground.
		Likely	2	Some risk of flooding, restricted access, heavily sloping or broken ground;
		Unlikely	3	High risk of flooding, no direct access, unsuitable ground foundation.
*Σ*	1.000	–	–	–

**Fig. 3:**
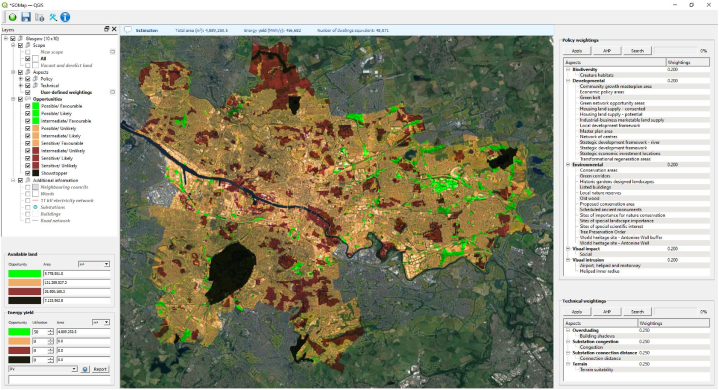
GOMap applied to Glasgow City where areas highlighted in green represent policy and technical unconstrained land for solar PV deployment.

The final outcome is presented as an opportunity map showcasing suitable locations for RET deployment as shown in Fig. 3 with all spatial information stored in the Selection Panel (top-left), and the weightings for each policy and technical aspect listed (top-right). Individual factors can be disabled, and aspect weightings modified to investigate other energy planning approaches. The amount of available land area highlighted by the opportunity map is transferred to the solar PV model that converts this into an annual energy yield, the number of PV arrays, and the amount of dwellings that can be supplied. The opportunity map can be scoped to focus on specific areas such VDL sites. The solar PV model generates polygon features based on optimal parameters and PV dimensions taking into consideration the site geometry and land utilisation. Knowing the exact amount of potential solar PV panels that can be installed allows for the annual energy yield of the site to be calculated. The number of dwellings supplied can then be inferred from the energy yield.

### 4.2 Verification

The GOMap tool was assessed independently by a Development planning manager at Glasgow City Council, verifying its output [34]. This assessment was based on a ‘Housing Proposal Project’ as part of the City Development Plan for additional housing opportunities in an area north of the city [73]. The spatial planning manager noted that GOMap's opportunity map reflected the Housing Project's Examination Report, which highlighted areas with policy and technical constraints. However, certain information not included in GOMap's assessment was included in the Examination Report, such as noise protection areas, traffic management, and air pollution risk. The spatial information for these factors was unavailable at the time. Although GOMap did not contain specific policy and technical factors which aligned with the Examination Report, it provided additional insight into the effects of other factors on site suitability that were not covered by the Report.

Expert verification enhances the credibility of GOMap's analytical capabilities and minimizes uncertainties in the final output.

### 4.3 Validation

The optimal solar PV parameters for Glasgow were determined through an inter-model comparison [34] with PVsyst, a commercial tool used widely in industry due to its accuracy [74]. This comparison involved GOMap's solar PV model and the PVsyst model calculating the potential annual energy output for a single 2 m × 1 m monocrystalline PV panel in Glasgow city for varying panel tilt angles, from 0^o^ to 90^o^ at an azimuth angle of 180^o^ with the exclusion of horizon brightening as this component required a paid license for PVsyst. The results had shown that GOMap and PVsyst calculated a maximum energy output of 159.2 and 158.9 kWh/m^2^.y respectively at a panel tilt angle of 40^o^. This marginal difference of 0.3 kWh/m^2^.y had concluded there was good agreement between the two models. A further iterative study was conducted which examined the maximum energy output at varying panel tilt angles at varying southern azimuth angles, from 135^o^ to 225^o^. Although the azimuth angle facing 180^o^ (due south) may be considered reasonable for solar PV installation in the northern hemisphere, the GOMap model uses equations of solar geometry and solar irradiance to provide an annual average energy yield which varies with panel tilt and azimuth and includes horizontal brightening. The optimal solar PV parameters for Glasgow, when the energy yield is at its maximum, are provided in Table 4.

**Table 4:** Optimal PV parameters for Glasgow [34].

PV parameter	Value
*Panel area*	2 m^2^
*Panel azimuth*	205^o^
*Panel tilt angle*	37^o^
*Array spacing*	7.4 m
*Energy yield*	172.8 kWh/m^2^.y

### 4.4 Scenario result and discussion

Once verified and validated, GOMap was used to evaluate the feasibility of constructing solar PV panel arrays on VDL throughout Glasgow using information from 2020 where the results were previously published [34,35].

GOMap will now be applied to Glasgow in the context of utilising VDL sites for solar PV deployment, and of utilising southerly-facing rooftops for BIPV deployment, using available information up to the year 2023.

Recent records show that Glasgow contains roughly 314,500 occupied dwellings [75] of which 107,000 (34 %) [76] are socially owned and, of these, 26,000 (24 %) lack any form of wall insulation [77] and categorised as ‘hard-to-heat’. The overall energy demand of a typical dwelling across Glasgow was 14,000 kWh/y [78,79], and as 75 % of this energy is used for space heating [80], the heating demand for a typical Glasgow dwelling was 10,500 kWh/y. From the given values, the City's overall domestic heating requirement was calculated to be approximately 3,300 GWh.

Four scenarios were devised to explore options for future improvements. Outcomes were compared the Scottish Government's Energy Strategy to electrify home heating by matching solar PV output with the energy needed to heat dwellings [81]. Table 5 presents the parameters for various scenarios, with certain policy and technical aspects disabled to examine the impact on unconstrained area availability and energy production. The results from these scenarios are tabulated in Table 6.

**Table 5:** Scenario information.

Scenario	Policy aspects and weightings	Technical aspects and weightings	Description
1	Biodiversity (0.326)	Overshading (0.484) Substation congestion (0.168) Substation connection distance (0.231) Terrain (0.117)	Base-case scenario with all policy and technical aspects active and weighted.
	Developmental (0.114)		
	Environmental (0.326)		
	Visual impact (0.148)		
	Visual intrusion (0.086)		
2	Biodiversity (0.429)	Overshading (0.484) Substation congestion (0.168) Substation connection distance (0.231) Terrain (0.117)	Future community education programs delivered and glare from solar PV disregarded.
	Developmental (0.168)		
	Environmental (0.429)		
3	Biodiversity (0.326)	Overshading (0.750) Terrain (0.250)	Future electricity infrastructure upgrades minimising congestion and overloading.
	Developmental (0.114)		
	Environmental (0.326)		
	Visual impact (0.148)		
	Visual intrusion (0.086)		
4	None	None	Retrofit BIPV measures disregarding land-based aspects.

**Table 6:** GOMap results for Glasgow.

Scenario	Area (ha)	Output energy (MWh/y)	No. of dwellings equivalent	Hard-to-heat dwellings equivalent
1	94.5	57,598	5,486 (±0 % base-case)	21 %
2	109.9	67,038	6,385 (+16 % of base-case)	25 %
3	183.9	111,938	10,661 (+94 % of base-case)	41 %
4	58.2	31,560	3,006	12 %

#### 4.4.1 Scenario 1

**Fig. 4:**
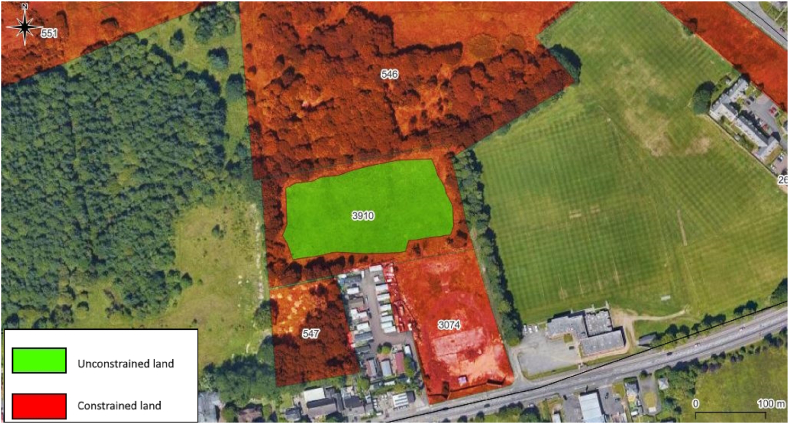
Site 3910 identified in Scenario 1.

The base-case Scenario 1 was configured with all aspect information active and weighted based on their perceived importance. For each of the policy aspects, the weightings (in parenthesis) were as follows: biodiversity (0.326), developmental (0.114), environmental (0.326), visual impact (0.148), and visual intrusion (0.086). For each of the technical aspects, the weightings (also in parenthesis) were as follows: overshading (0.484), substation congestion (0.168), substation connection distance (0.231), and terrain (0.117). Evaluating this information revealed 94.5 ha of unconstrained VDL with an energy potential of 57,598 MWh/y. This could supply up to 5,486 of Glasgow's dwellings or 21 % of hard-to-heat dwellings. Fig. 4 shows a site in Scenario 1 where areas shown as green are designated as unconstrained in terms of both policy and technical; and areas shown as red are designated as constrained. The numbers in the figure are the Site IDs assigned by Glasgow City Council.

#### 4.4.2 Scenario 2

Scenario 2, with two policy aspects relaxed and all technical aspects active, explores the idea of encouraging communities to weigh the benefits of solar PV arrays more than the potential detriment of the local aesthetic. Additionally, glare from PV affecting pilots is disregarded due to the lack of evidence suggesting such phenomenon poses a hazard. The weightings for the remaining three aspects were recalculated: biodiversity (0.429), developmental, and environmental (0.429). The results identified VDL sites equating to an area of 109.9 ha capable of generating energy equivalent to heating 6,385 dwellings, an increase of 16 % from the base-case scenario and equivalent to 25 % of hard-to-heat dwellings.

#### 4.4.3 Scenario 3

Scenario 3, with all policy aspects active and two technical aspects relaxed, explores the idea of resources made available to create new and improved energy infrastructure. The weightings for the remaining two aspects were recalculated: overshading (0.750), and terrain (0.250). This results in 183.9 ha of VDL sites which can supply 10,661 dwellings or 41 % hard-to-heat dwellings. This scenario can provide for 67 % more dwellings than Scenario 2 and 94 % more than Scenario 1.

#### 4.4.4 Scenario 4

Scenario 4 detected dwellings with southerly-facing rooftops for BIPV retrofit. As no policy or technical information was available for individual buildings (permission for BIPV lie with the homeowners or local housing associations), all policy and technical aspects were relaxed. Instead, other considerations were considered such as the dwellings must be occupied and there must be a minimum of 20 m^2^ roof space to give adequate coverage [82]. Spatial information contained within a 2 m resolution DSM and building polygon shapefile were provided by the Centre of Environmental Data and Ordnance Survey respectively [83,84]. From a total of 314,500 inhabited dwellings, 3.7 % or 11,530 dwellings were identified with south-facing rooftops. This results in a combined roof area of 58.2 ha which can be translated into 31,560 MWh/y, the equivalent of 3,006 dwellings which amounts to 12 % of the hard-to-heat stock. Alternatively, if all 11,530 dwellings proceeded with BIPV retrofit, 2.74 MWh could potentially be provided to each dwelling which is equivalent to 26 % of its average annual heating demand.

**Fig. 5:**
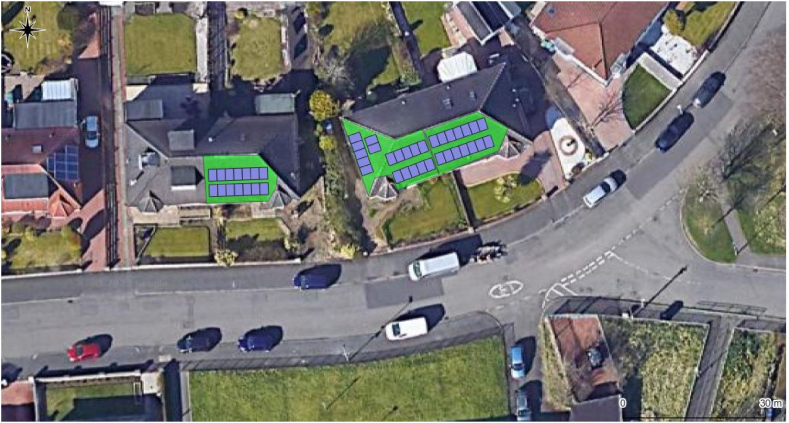
Example of script identifying south-facing rooftops represented by green polygons, and its area passed to the in-built solar PV model with PV panels represented by blue polygons.

Fig. 5 shows three dwellings with rooftops facing in the southerly direction. GOMap generates solar PV panels with specifications of 2 m × 1 m dimensions, all panels are enclosed within the perimeter of the roof. Comparisons can be made between existing panels installed on a rooftop shown in the satellite imagery, and panels generated by GOMap and where the former panes are installed on the east side of the dwelling and lacking direct sunlight at certain times of the day, and the latter panels are superimposed with suitable south-facing orientation and exposed to sunlight most of the day.

## 5 Conclusions

The study aimed to develop and test a geospatial site selection method for local energy generation that integrates policy and technical aspects. The development process involved workshops with local authorities and utility personnel to address policy and technical aspects, aiming to meet local communities' energy demands, reduce carbon emissions, and ensure greater energy security. Three systems were developed for evaluating policy and technical factors in solar PV deployment: a grid system for spatial relationships, a scoring system for distinguishing between factors supporting or limiting deployment, and a weighting system for prioritizing each aspect. These systems and all policy and technical information are encapsulated in the freely available GOMap tool. Once potential sites are identified, these are exported to a solar PV array evaluation tool to estimate the annual energy yield based on hourly weather information and design parameters such as panel azimuth, tilt angle and inter-row spacing generated automatically or input by the user.

The results are communicated via an opportunity map with the intention that GOMap can assist stakeholders and decision makers to collaborate toward the goal of embedding effective RET solutions within urban environments. The tool doesn't address integration with commercial development, costs, or technical design, nor does it eliminate the need for normal planning control for new developments as the accuracy of the tool is not only dependent on the input data but can also project subjective bias as users can change factor scores and aspect weightings without due process. Therefore, robust procedures should be developed to ensure high accuracy is maintained throughout the process.

**Fig. 6:**
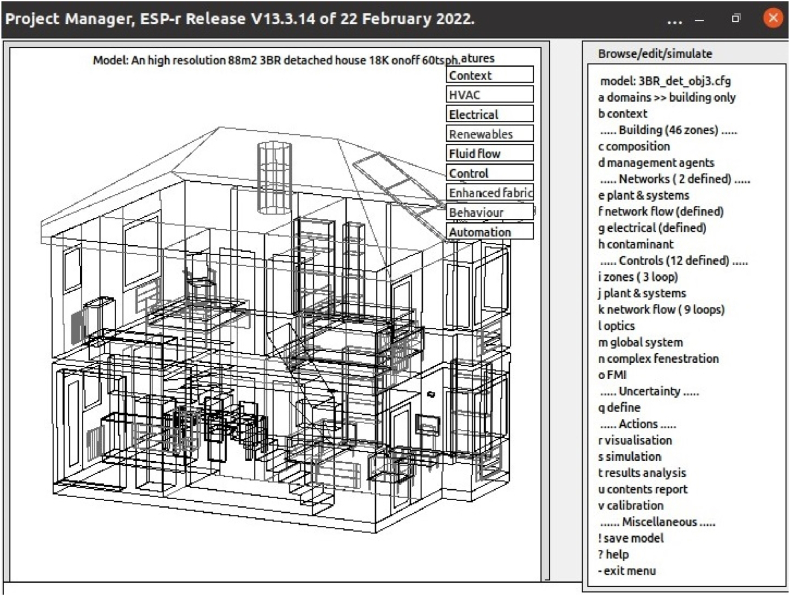
ESP-r interface with a project loaded.

There have been significant developments in energy systems performance simulation [85]. ESP-r is an example that supports dynamic analysis of buildings when serviced from conventional and renewable energy resources [86,87]. The system (Fig. 6) can assess the energy and indoor/outdoor environmental impact of any proposed design [88] to enable a deeper analysis of solar PV community impact. For example, as suitable sites are identified in GOMap, ESP-r can access relevant weather information and execute simulations to obtain optimal PV parameters. Where the ESP-r model also includes a representation of surrounding buildings and the low voltage network connections, it is possible to explore the matching of supply and demand over any period and the impact on this match of smart control strategies.

In the future, GOMap will be integrated with the ESP-r urban energy simulation program to determine community electrical requirements, considering occupancy behaviour and smart control systems [89]. Populating GOMap with such information will enable the creation of city maps depicting the match between energy demand and supply at the community level.

## Data availability

The GOMap tool, its source code and the data used are available from:

ESRU repository: https://www.esru.strath.ac.uk/applications/gomap.

ESRU GitHub: https://github.com/ESRU-Strathclyde/GOMap.

## CRediT authorship contribution statement

**R. McGhee:** Writing – review & editing, Writing – original draft, Visualization, Software, Methodology, Investigation, Formal analysis, Data curation. **J.A. Clarke:** Supervision, Project administration, Investigation, Funding acquisition, Conceptualization. **K. Svehla:** Visualization, Resources, Methodology, Investigation, Formal analysis, Data curation.

## Declaration of competing interest

The authors declare that they have no known competing financial interests or personal relationships that could have appeared to influence the work reported in this paper.
